# Spheroid Formation and Peritoneal Metastasis in Ovarian Cancer: The Role of Stromal and Immune Components

**DOI:** 10.3390/ijms23116215

**Published:** 2022-06-01

**Authors:** Militsa Rakina, Anna Kazakova, Alisa Villert, Larisa Kolomiets, Irina Larionova

**Affiliations:** 1 Laboratory of Translational Cellular and Molecular Biomedicine, National Research Tomsk State University, 634050 Tomsk, Russia; militsarakina@mail.ru (M.R.); ms.anya.kazakova.2015@mail.ru (A.K.); 2 Department of Gynecologic Oncology, Cancer Research Institute, Tomsk National Research Medical Center, Russian Academy of Sciences, 634009 Tomsk, Russia; avillert@yandex.ru (A.V.); kolomietsla@jncology.tomsk.ru (L.K.); 3 Laboratory of Cancer Progression Biology, Cancer Research Institute, Tomsk National Research Medical Center, Russian Academy of Sciences, 634009 Tomsk, Russia

**Keywords:** ovarian cancer, spheroid, composition, malignant ascites, peritoneal metastasis, tumor-associated macrophages, cancer-associated fibroblasts, T-lymphocytes

## Abstract

Ovarian cancer (OC) is one of the most common gynecological cancers, with the worst prognosis and the highest mortality rate. Peritoneal dissemination (or carcinomatosis) accompanied by ascites formation is the most unfavorable factor in the progression and recurrence of OC. Tumor cells in ascites are present as either separate cells or, more often, as cell aggregates, i.e., spheroids which promote implantation on the surface of nearby organs and, at later stages, metastases to distant organs. Malignant ascites comprises a unique tumor microenvironment; this fact may be of relevance in the search for new prognostic and predictive factors that would make it possible to personalize the treatment of patients with OC. However, the precise mechanisms of spheroid formation and carcinomatosis are still under investigation. Here, we summarize data on ascites composition as well as the activity of fibroblasts and macrophages, the key stromal and immune components, in OC ascites. We describe current knowledge about the role of fibroblasts and macrophages in tumor spheroid formation, and discuss the specific functions of fibroblasts, macrophages and T cells in tumor peritoneal dissemination and implantation.

## 1. Introduction

Ovarian cancer (OC) is the third most common malignant gynecological disease after cervical and endometrial cancers [1]. Among gynecological malignancies, OC is characterized by the worst prognosis and the highest mortality rate [2,3]. Research on ovarian cancer is currently focused on a large number of unresolved issues associated with diagnosis at the late stages, difficulties in treatment strategy selection, the high proportion of relapses, low treatment efficacy and high mortality rates.

The lack of methods for early detection of OC is a major obstacle, leading to a high rate of diagnoses with advanced-stage (III-IV) disease that hinders therapy efficiency [4]. The principal hallmark of advanced-stage OC is ascites—the accumulation of excessive fluid containing cellular and acellular components in the abdomen [5].

According to the International Federation of Gynecologists and Obstetricians (FIGO), the overall five-year survival rate is approximately 50%, but this could rise to 90% for stage I–II ovarian cancer when the tumor is within the ovary [6,7]. The five-year survival rate for patients with disseminated tumors (stages III–IV) is only about 20% or lower [7]. Despite a good first-line response to standard platinum/taxane-based chemotherapy (cisplatin/carboplatin and paclitaxel/docetaxel), multiple drug resistance-associated relapse occurs in 70% of patients within a short period of time [8,9]. Another distinctive feature of OC is a transcoelomic route of metastasis, which is more frequent than standard hematogenous/lymphatic paths and is observed in about 70% of patients [5,10]. Peritoneal (trancoelomic) metastasis development is related to the dissemination of tumor cells after their detachment from the primary tumor and movement through the ascitic fluid to the most common metastatic sites, i.e., the omentum and parietal and visceral peritoneum, as well as direct implantation to adjacent organs, resulting in increased mortality of OC patients [5,10]. Lymphatic metastasis usually occurs in pelvic and para-aortic lymph nodes. Hematogenous spread is the least common metastatic path [11,12].

Ascites (from the Greek “askos”—bag, sack) is a condition characterized by an abnormal accumulation of fluid in the abdominal cavity [13]. A relationship between OC stage and the volume of ascitic fluid has been shown. The incidence of ascites was 49.4% in stage I and 62.5% in stage II disease, increasing to 90.1% and 100% in stages III and IV, respectively [14]. The mechanism of ascites formation, complicated by peritoneal dissemination (carcinomatosis), is complex [15]. During tumor progression, the cross-sectional area of the microvessels lining the abdominal cavity is enlarged, causing excessive fluid filtration [11]. Secondly, the concentration of proteins in malignant ascites increases, and the tumor overproduces the most critical pro-angiogenic factor—vascular endothelial growth factor (VEGF) [15,16]. The tumor initiates a pro-inflammatory response in the abdominal cavity, thereby promoting the attachment of tumor cells to the surface of the peritoneum and intraperitoneal organs [17]. The obstruction of lymphatic vessels by tumor cells leads to a violation of ascitic fluid reabsorption and an increase in lymph viscosity. Thus, retrograde lymph flow can accelerate ascites formation, along with some anatomical and physiological factors, such as gravity, intradiaphragmatic pressure and mobility of internal organs [10,18,19].

Peritoneal dissemination (or carcinomatosis) is the most unfavorable factor in the progression and recurrence of malignant tumors in the organs of the peritoneal cavity [20]. The frequency of peritoneal dissemination in primary OC is alarmingly high, and is comparable to the proportion of diagnosed advanced forms. This actually limits the effectiveness of surgical and chemotherapeutic anti-cancer treatments. The severity of carcinomatosis in OC is directly related to the neglect of the disease and the production of ascitic fluid [18].

Due to the availability of ascitic fluid for research, a number of recent studies have focused on the analysis of the diagnostic and prognostic significance of individual markers in peritoneal fluid. However, there is still no clear idea about the composition of tumor spheroids, the quantitative and qualitative composition of the cellular component of tumor spheroids and the ascites itself in disseminated forms of ovarian cancer. In the present review, we provide data on the main cellular compounds of ascites and tumor spheroids, i.e., tumor-associated macrophages, cancer-associated fibroblasts and T-lymphocytes. We discuss possible mechanisms of spheroid formation, focusing on their prognostic significance for ovarian cancer dissemination.

## 2. Cellular Composition of Ascites and Spheroid Formation

Malignant ascites (MA) contains cellular (tumor cells, diverse immune cells, fibroblasts, adipocytes, mesothelial cells, and extracellular microvesicles) and acellular (cytokines, growth factors and lipid mediators) components. The latter ensures the interaction among cellular elements [21]. There is a lack of quantitative data on the cell composition of ascitic fluid. In one report, the tumor cell population, defined as CD45-EpCAM+, fluctuated from 1 to 85% in ascites samples, whereas the range of immune and mesenchymal-like cells was between 10–30% [22]. Another study revealed that the total cellular population in ascitic fluid is comprised of 37% lymphocytes, 29% mesothelial cells, 32% macrophages and also very few (<0.1% of the total) adenocarcinoma cells [23].

**Figure 1:**
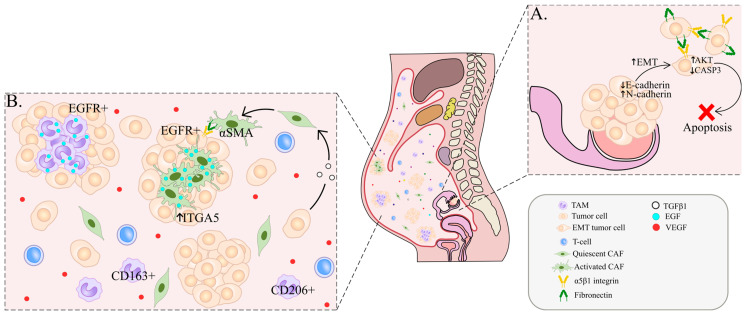
Tumor cell survival and spheroid formation in malignant ascites. (**A**). Downregulation of E-cadherin and upregulation of N-cadherin leads to EMT and a loss of cell–cell contact between tumor cells, followed by their detachment from the primary site. In order to evade anoikis, tumor cells form clusters through α5β1 integrin and fibronectin interactions. Activation of AKT leads to inhibition of caspase-3, which also hinders apoptosis. (**B**). One of the possible mechanisms of spheroid formation is mediated by the EGF-EGFR axis between TAMs/CAFs and tumor cells. Fibroblasts secrete EGF that upregulates *ITGA5* expression in tumor cells as well as *TGFβ*, leading to strengthened tumor–stromal interaction inside spheroids. In turn, CAFs activated by *TGFβ*- and *ITGA5*-overexpressing tumor cells secrete EGF and other TGFβ-associated factors. EGF+ TAMs induce EGFR+ tumor cell migration and spheroid formation. Free-floating TAMs abundantly express CD206 and CD163 in ascites. A major marker for CAFs is α-SMA. VEGF is a key factor in ascitic TME.

The cellular component of ascites can be divided into so-called “resident cells”, such as tumor cells and cancer-associated fibroblasts, or stromal cells, and “non-resident cells”, such as immune cells and mesenchymal stem cells [24]. The processes of auto/paracrine communication and reciprocal interactions between the stromal component of the tumor microenvironment (TME), tumor stem cells and growing tumor foci induce a pro-inflammatory response, correlating with the number of various autocrine and paracrine molecules (growth factors, cytokines, chemokines, matrix proteases, immunosuppressive factors) potentiating tumor growth (Figure 1) [5].

The cellular components of MA can be either in a free-floating state or form spheroids, leading to intraperitoneal metastases [11,25,26]. Spheroids can vary in size and structure, and can be composed solely of tumor cells or contain stromal and immune cells [15]. The question regarding the similarity between the molecular genetic characteristics of free-floating tumor cells in ascitic fluid and cells constituting solid tumors remains open. These cells are phenotypically different and, according to the concept of clonal evolution, should reflect different stages of tumor progression [27]. However, a comparison of expression profiles in patients with primary and metastatic tumors showed that ascites samples retained the molecular diversity of primary tumor samples and, therefore, could be fully used for investigations into novel therapeutic approaches [28,29]. Many reports have been published on the usefulness of growing tumor cells from ascites in vitro to perform drug susceptibility tests and predict clinical resistance by assessing biomarkers in ascites [30,31]. In vitro, spheroids have been shown to limit both the access of chemotherapeutic agents to tumor cells and the efficacy of classical cytotoxic drugs [32,33]. The spheroid population was found to be four times more resistant to cisplatin compared to a single-cell OC population [15].

## 3. Peritoneal Metastasis

Peritoneal dissemination is the main factor determining tumor resectability, and is an unfavorable parameter for survival rates in patients with advanced or recurrent OC [34]. The first key process in transcoelomic metastasis is the rupture of the capsule containing the primary tumor. This allows cancer cells to disseminate after their detachment from the primary tumor site and form spheroids [10,35]. The initial implantation sites are the fallopian tubes and contralateral ovary [36]. After that, the most common metastatic sites are the omentum, the parietal and visceral peritoneum, as well adjacent organs via direct implantation [10,36]. The current scenario of intraperitoneal dissemination in OC includes tumor cell proliferation and epithelial-mesenchymal transition (EMT), the latter of which results in tumor cell migration, and, conversely, mesenchymal epithelial transition (MET), which forces the colonization of tumor cells with the formation of peritoneal implants [5,15,37,38].

Omental milky spots (MSs) are the major implantation site for malignant cells during peritoneal dissemination [39]. MSs are small, capsule-free specific structures, consisting of macrophages, lymphocytes, blood and lymphatic vessels, which enable fluid exchange between the peritoneal cavity, the blood stream and the adjacent omental tissue [39,40,41]. The important role of MSs in tumor cell dissemination, attachment, invasion and proliferation within MSs has been extensively studied in vivo [42,43]. In orthotopic ovarian cancer models, OC cells invaded mice omental MSs within minutes after intraperitoneal injection [44,45].

**Figure 2:**
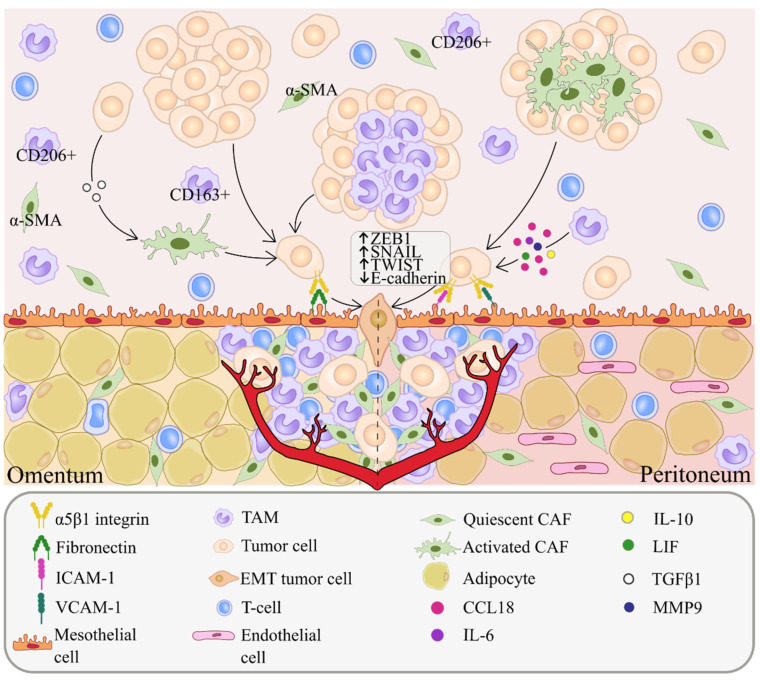
Mechanisms of implantation metastasis. α5β1 integrin on tumor cells binds to exposed fibronectin on mesothelial cells, facilitating the development of peritoneal metastasis. The morphology of implanted tumor cells resembles that of mesenchymal cells with increased expression of mesenchymal markers (ZEB1, SNAIL and TWIST) and decreased E-cadherin expression during invasion to the omentum/peritoneum. TAM-derived CCL18 promotes EMT in tumor cells. IL-10, IL-6, MMP9 and LIF produced by TAMs enable the implantation of tumor cells. TGFβ-activated fibroblasts induce the adhesion of tumor cells to a layer of mesothelial cells.

Single floating tumor cells in ascites undergo anoikis, an apoptosis induced by the lack of proper cell/extracellular matrix (ECM) attachment [46]. Increased survival of tumor cells can be achieved by the formation of multicellular spheroids in the peritoneal cavity containing immune and mesenchymal cells, among with tumor cells [36]. AKT kinase is activated in clustered OC cells, and stimulates their survival through inhibition of caspase-3, an apoptosis regulator (Figure 1A) [47]. The development of peritoneal carcinomatosis is a multistep process that requires tumor adhesion and growth. Related mechanisms involve cadherin reorientation on ovarian cancer cells, integrin-mediated adhesion and mesothelial evasion by integrin-ligand interactions [47]. The detachment and subsequent formation of tumor cell clusters in MA are associated with downregulation of E-cadherin expression. OC cells express integrins, i.e., glycoproteins that contribute to the adhesion of tumor cells to the peritoneum (Figure 1A) [48]. Among integrins, α5β1 integrin is abundantly expressed in OC cells and binds to exposed fibronectin on mesothelial cells. This interaction facilitates the development of peritoneal metastases. Ligands of integrins also include collagen, laminin, fibrinogen, vitronectin, intercellular adhesion molecules (ICAM)-1 and vascular cell adhesion molecules (VCAM)-1 (Figure 2) [35,47].

Additionally, VEGFs stimulate the vascular and lymphatic endothelium to form new blood vessels to support the growth of ovarian cancer cells and spheroids, as well as MA formation (Figure 1B) [34,35]. Several studies have revealed the interplay between VEGF and MMPs during the peritoneal spread of ovarian cancer. Ascitic fluid from OC patients containing high levels of MMP-2 and VEGF induced an increase in the invasive and angiogenic activity of tumor cells in vitro [49]. In nude mice bearing human ovarian carcinoma xenografts, MMP-9 levels correlated to VEGF release and ascites formation [49]. In the early stages of carcinogenesis, VEGF and its receptors are the main drivers of angiogenesis in the tumor, but in the process of tumor progression, other pathways are activated, leading to the development of bevacizumab (anti-VEGF drug) resistance [16,50,51]. Interestingly, the presence of ascites may be a predictor of improved efficacy of bevacizumab therapy [52,53].

## 4. Cancer-Associated Fibroblasts (CAFs)

Cancer-associated fibroblasts (CAFs) play a pivotal role in tumor progression. CAFs enhance cancer cell proliferation [54], angiogenesis and lymphangiogenesis [55], ECM remodeling, immune cell recruitment [56], invasion and metastasis via cytokine and chemokine secretion [57,58,59,60,61,62]. CAFs are a highly heterogeneous subpopulation of stromal cells in TME, originating from different precursors, including resident tissue fibroblasts, bone marrow mesenchymal stem cells, hematopoietic stem cells, epithelial cells and endothelial cells [63,64,65].

In ascites, CAFs originate from recruited fibroblasts and mesothelial cells [65]. Here, CAFs usually exist as free-floating cells and rarely as a part of spheroids [66,67]. Free-floating CAFs, as well as TAMs, form the ecosystem of ovarian cancer ascites and provide a suitable microenvironment for cancer progression [66]. Single-cell RNA-seq of ascites samples from high-grade serous ovarian carcinoma (HGSOC) patients revealed subpopulations of CAFs expressing immune-related genes that were categorized as complement factors (*C1QA/B/C*, *CFB*), chemokines (*CXCL1/2/10/12*) and cytokines (*IL6* and *IL10*), which are responsible for the activation of JAK/STAT signaling in tumor cells. Inhibition of JAK/STAT reduced the formation of spheroids and their invasion through a mesothelial monolayer in vitro, and decreased the development of malignant ascites and tumor growth in a patient-derived xenograft model in vivo [68].

CAFs in ascites are involved in intercellular interactions with stromal cells and floating tumor cells through mechanisms similar to the ones in tumor tissue: cytokine secretion, ECM remodeling and immune cell recruiting [68,69,70,71]. Cancer cells can interact with stromal cells, such as fibroblasts and macrophages, and form heterotypic spheroids in ascites [69]. CAFs form the core of such spheroids and can serve as a scaffolding to aggregate floating tumor cells [54,69,72]. Immunofluorescent analyses revealed that ascites spheroids contain EpCAM+ epithelial cells surrounding the CAF core, that is mainly characterized by α-smooth muscle actin (α-SMA) expression, or platelet-derived growth factor receptor-β (PDGFRβ), prolyl 4-hydroxylase, FSP-1 and FAP positive staining (Figure 1B) [54,69]. Several in vitro studies confirmed that heterospheroids are more invasive, display the lowest apoptosis rate in suspension culture (anoikis), enhanced adhesion to mesothelium and are more resistant to chemotherapeutic agents than homospheroids [54,69,70,72,73]. Due to their high malignant potential and contribution to peritoneal dissemination, such heterospheroid structures are referred to as metastatic units [69]. Surprisingly, ovarian cancer cells in spheroids can express fibroblastic marker αSMA and fibronectin (FN1), which are associated with EMT. The same cells inside spheroids maintain the expression of epithelial marker, EpCAM, indicating that ovarian cancer cells in spheroids may undergo so-called “partial” EMT or “epithelial-mesenchymal plasticity” [66,74].

CAFs contribute to compact spheroid formation via the activation of cadherins and intergrins, which play the most significant role in cell–cell interactions [65,74,75]. High E-cadherin expression is associated with large, more spherical cells which grow in aggregates compared to small, polygonal or spindle-shaped distributed cells with few or no E-cadherin expression. Cells with high E-cadherin expression demonstrate tighter cellular connections in suspension, resulting in the formation of compact multicellular spheroids with longer lifespans [76]. An in vitro co-culture of primary OC cells, derived from serous epithelial OC effusions, and mesothelial cells, obtained from peritoneal lavage of women with benign gynecological conditions, was established for spheroid formation. In this model, β1-integrin was the main cell–cell interaction molecule contributing to compact spheroid formation, whereas the expression of MUC16 and E-cadherin was associated with the formation of loose and much less compact spheroids [65]. CAFs secrete epidermal growth factor (EGF) that upregulates integrin α5 (*ITGA5*) expression on tumor cells, as well as TGF-β1, leading to strengthened tumor–stromal interactions inside malignant units. In turn, CAFs activated by ITGA5-overexpressing tumor cells secrete TGFβ-associated factors EGF, IP-10, IGFBP-3, BDNF, Flt-3 LG, FGF-7, IL-12, MIF and leptin (Figure 1B) [69]. Multicellular spheroids containing TGF-β1-activated fibroblasts were shown to be smaller in comparison to non-activated spheroids due to the presence of a denser ECM. Fibroblast activation resulted in increased collagen deposition followed by ECM stiffness [70]. The formation of compact spheroids with stiffened ECM is a major obstacle to therapy efficacy that mediates enhanced integrin-mediated pro-survival signaling and the high degree of invasiveness of tumor cells [74].

In addition to the organization of spheroids, fibroblasts are actively involved in the formation of pre-metastatic niches, the promotion of peritoneal adhesion and the implantation of tumor cells [77,78]. Malignant ascites contains various metastasis-promoting mediators, produced by both tumor cells and CAFs, such as TGF-β1, HGF, GRO-1 and IGF-1. Tumor cells are able to activate peritoneal fibroblasts through TGF-β1 secretion [70,73,78,79,80]. In an in vitro study, TGF-β1 was found to lead to the activation of mesothelial–mesenchymal transition in human peritoneal mesothelial cells, their transformation into fibroblasts and fibrosis and the creation of a favorable microenvironment for tumor cell dissemination [79]. Omental co-culture models consisting of SCOV3 cells cultured with CAFs, normal omental fibroblasts and TGF-β1-activated fibroblasts were established. These models showed that CAFs and activated fibroblasts induce stronger adhesion of tumor cells to a layer of mesothelial cells [73]. There was an upregulation of HGF and MMP-2 in activated fibroblasts and CAFs compared to the control, suggesting their key role in the adhesion of tumor cells [73]. An in vitro cell adhesion assay showed that tumor cells demonstrate stronger TGF-β1 and IGF-1-related adhesion to peritoneal cells in the presence of malignant ascites [80]. α5β1 integrins interact with ECM in cooperation with integrin-linked kinase (ILK). An immunofluorescent analysis showed that TGF-β1 and IGF-1 upregulate the expression of other adhesion-associated molecules, such as ICAM-1 and vimentin, on the surface of ascites-treated tumor cells and peritoneal cells [80].

## 5. Tumor-Associated Macrophages (TAMs)

As suggested by several studies, macrophages are the most predominant population of immune cells in ascites, constituting up to 95% of MA cellular components [21,22,25,68,81]. Peritoneal macrophages play an essential role in the suppression of inflammation and the regulation of immune response in physiological and pathological conditions [82]. They are present in the peritoneal cavity of healthy women, but their number increases in advanced EOC ascites [83].

In malignant ascites, TAMs float separately or are located in the center of spheroids surrounded by tumor cells; they possess M2 polarization by the abundant expression of CD163 and CD206 [83,84,85]. Additionally, fewer CD163+ TAMs can be found outside of spheroids in a free-floating state [66].

TAMs also protect ovarian cancer cells from anoikis by inducing the secretion of several soluble factors which promote the peritoneal dissemination of tumor cells and support their proliferation via STAT3 signaling [5,83,86,87]. In an orthotopic mouse model of ovarian cancer, macrophages promoted spheroid formation and induced the proliferation of free-floating tumor cells in ascitic fluid [84]. In this model, the number of infiltrating F4/80+, CD11b+ and CD68+ macrophages increased drastically 8 weeks after injection of tumor cells into the peritoneal cavity, and macrophages displayed M2-polarization markers (by the expression of *CD163*, *CD206* and *CX3CR1*). Within the large spheroids, EGFR+ tumor cells surrounded the central EGF+ macrophages. Mechanistically, EGF secreted by TAMs induced EGFR+ tumor cell migration and TAM spheroid formation through VEGF-C/VEGFR3 signaling (Figure 1B). EGF facilitated the adhesion of EGFR+ tumor cells with TAMs through ICAM-1–αMβ2 integrin interaction. EGFR blockade using erlotinib decreased the amount of TAM and inhibited spheroid formation and OC progression in vivo. In ovarian cancer patients, the five-year overall survival rate was significantly lower in OC patients with high percentages of CD68+ TAMs (>14.5%) in spheroids compared with low percentages (<14.5%) of CD68-positive cells [84]. EGF-secreted TAMs also increased the invasive and migratory capacity of SKOV3 cells isolated from in vitro 3D spheroids, generated by TAMs and tumor cells [85]. Spheroid formation was facilitated by the CCL18-ZEB1-M-CSF axis. TAM-derived CCL18 induced EMT in tumor cells. The morphology of SKOV3 cells isolated from spheroids resembled that of mesenchymal cells with increased expression of the mesenchymal markers (ZEB1, SNAIL and TWIST) and decreased E-cadherin expression, compared to SKOV3 cells in a transwell system (Figure 2). In vivo, spheroids containing ZEB1-overexpressed OC cells and TAMs were shown to be critical for transcoelomic metastasis [85].

TAMs also promote endothelial permeability in ascites. Human CD33+CD68+MHCII−CD206+ M2 macrophages, isolated from OC patient ascites, and MHCII-negative M2 macrophages, isolated from murine malignant ascites, induced vascular dysfunction in a VEGF-independent manner [88]. Since TAMs are the major source of diverse pro-angiogenic factors in TME, they can regulate EC functions by involving different angiogenic pathways [16]. In vivo macrophage blockade by CSF1R inhibitor resulted in a reduction of macrophage number in the ascites and vascular normalization [88]. In an orthotopic ovarian cancer model, apoptosis signal-regulating kinase 1 (ASK1) regulated EC permeability in the peritoneal cavity and macrophage transmigration to ascites by regulating EC junctions [89]. In vivo ASK1 deficiency decreased the amount of CD68^+^ macrophages inside the spheroids but not the polarization of TAMs, attenuating TAM-spheroid formation and tumor peritoneal implantation [89].

Numerous soluble tumor cell-derived and TAM-derived factors in ascites facilitate tumor progression [36,84,85,90]. A transcriptomic analysis revealed several signaling networks providing tumor cell-TAMs interactions in ascites. They involve STAT3-inducing cytokines (IL-10, IL-6 and LIF), TGFB1 mainly expressed by TAMs, WNT7A mainly expressed by tumor cells, multiple S100 genes, semaphorins and their receptors (plexins and neuropilins), ephrins, chemokines and their receptors [91]. The gene expression of *IL-10*, *TGFb1*, *S100A8*, *S100A9*, and *IL10RA* was increased in TAMs compared to tumor cells isolated from ascites of OC patients [91]. Several soluble mediators produced by ascites-derived TAMs, e.g., TGFß1 protein, tenascin C (TNC) and fibronectin (FN1), activated tumor cell migration [26]. Ascitic TAMs from OC patients express high levels of CCL18 [92], the immunosuppressive factor involved in cancer immune evasion (Figure 2) [93]. The CCL18 levels in the ascites of patients with serous OC were significantly higher compared to those in the peritoneal fluid of patients with benign gynecological conditions [94]. Cell free ascitic fluid containing CCL18 induced the migration of CaOV3 and OVCAR3 cancer cell lines in vitro in a dose-dependent manner [94].

A phenotypic analysis of TAMs in ascites of ovarian cancer patients revealed distinct macrophage subpopulations that possessed pro-tumor functions [95]. A flow cytometry analysis of TAMs isolated from the ascites of primary HGSOC showed the presence of both M1 macrophages (CD14+/CD80+/Glut1+) and M2 macrophages (CD14+/CD163+) [95]. Patients with high M1/M2 ratios (more than 1.4) had a significantly longer overall survival (OS), progression-free survival (PFS) and platinum-free interval than patients with low M1/M2. Patients with platinum-sensitive tumors showed a significantly higher M1/M2 ratio than those with platinum-resistant tumors [95].

A transcriptomic analysis of TAMs isolated from the ascites of OC patients revealed two signatures of expressing genes: A, characterized by the overexpression of pro-tumor and immunosuppressive markers (*CD163*, *PCOLCE2*, *IL6*) related to ECM remodeling; and B, with low expression of pro-oncogenic and immunosuppressive markers and increased regulation of genes associated with interferon signaling [96]. Signature A genes were strongly associated with a short OS, while signature B genes were significantly related to a favorable clinical outcome [96]. RNA sequencing of TAMs isolated from the ascites of patients with HGSOC demonstrated that CD163+ or CD206+ macrophages displayed increased expression of pro-tumor growth factors and cytokines, such as *CCL18*, *KITLG*, *SEMA6B*, *S100B* and *VEGFB*, and tumor suppressive mediators, e.g., *CXCL10*, *CXCL11*, *IL15*, *TNFSF10* and *TNFSF14* [97]. The increased expression of genes encoding the proteins involved in ECM (*ADAMTS2*, *CTSB*, *FBLN5*) and complement factors (*C1QC* and *CR1L*) has also been found in TAMs expressing CD163 or CD206. Additionally, TAMs from ascites produce chemotactic mediators CCL5, CXCL8, IL1RN, CCL18, CXCL2 and CXCL3, which are essential for monocyte/macrophage recruitment [97]. Using single cell sequencing of immune and tumor cellular components from the ascites of HGSOC patients, two distinct macrophage subpopulations were revealed [68]. Cells from the first subpopulation expressed genes of M1 polarization (*IFNGR1*, *CD36*, *DDX5*, *MNDA*), while the second subpopulation expressed genes related to M2 phenotype (*AIF1*, *VISG4*) [68]. Another interesting observation was the shift from the M1 to the M2 phenotype in macrophages in samples taken from a single patient before and after chemotherapy, which could suggest a role of neoadjuvant chemotherapy in macrophage polarization to malignant phenotypes; however, further studies need to be undertaken to support this theory [68].

## 6. T Cells

The activation of the immune system against tumor cells is expected to lead to a prolonged survival of cancer patients [98]. T lymphocytes play a critical role in the host immune system’s ability to eliminate tumor cells [99]. Three main subtypes represent T cells in ovarian cancer ascites: CD8+ effector cells, CD4+ helper cells and regulatory T cells (Tregs) [100,101]. These contribute to the activation and the regulation of immune response in OC patients [102]. In ascitic fluid, T cells are free-floating; there are no data on their contribution to spheroid formation (Figure 1 and Figure 2) [103,104].

Several studies have demonstrated correlations between ascitic T cells and clinical outcome in patients with HGSOC [103]. A high CD8/CD4 ratio in ascites was associated with significantly improved survival in OC patients [103]. Another study showed a trend toward improved PFS and OS in patients with low percentages of CD4+ T cells in ascites [101]. The accumulation of CD8+ effector memory T cells in ovarian cancer ascites was directly associated with a more favorable clinical outcome. The recruitment of CD8+ cells to the peritoneal effusion was accompanied by TAM-derived CXCL9 [104].

Tregs, which are abundantly represented in malignant ascites, promote tumor development and progression by inhibiting antitumor immunity [105,106,107]. A higher percentage of Treg cells in peripheral blood before chemotherapy correlated to worse long-term outcome in OC patients [108]. Tregs in ascites often demonstrate elevated expression of transcription factor FOXP3 [100,109], that stimulates PD-L1, a negative immunoregulatory molecule which inhibits effector T-cells [110]. Comparing malignant (ovarian cancer) and nonmalignant (idiopathic cirrhosis) ascites, the infiltration of Tregs was significantly higher in the former [106].

Tumor cells can escape the immune system by reducing T cell proliferation via secretion of lipid metabolites such as 5-HETE, 9-HODE and PGD2 [111]. The concentrations of these metabolites were significantly increased in OC patient ascites compared to normal abdominal fluid. PGD2, 5-HETE and 9-HODE were linked to peroxisome proliferator-activated receptor (PPAR) activation in T cells, resulting in the suppression of T cell proliferation through the STAT3/NFkB/cyclin E axis [111]. The in vitro cultivation of T cells in acellular ascites, isolated from OC patients, resulted in the suppression of IFN-γ, TNF-α, CCL4 and CD107a expression in lymphocytes and hindered their proliferation [112]. T cells from healthy donors stimulated by acellular OC ascitic fluid underwent IRE1α/XBP1-driven suppression of mitochondrial activity and IFN-γ production [113]. Abrogation of the IRE1α-XBP1 axis enhanced mitochondrial respiration in T cells. In an orthotopic OC model, the lack of XBP1 in T cells correlated to activated anti-tumor immunity, delayed disease progression and increased overall survival [113].

A comparative characterization of T cells in ascites, tumor mass and peripheral blood was performed in OC patients [100]. A transcriptomic analysis revealed pronounced differences in T cell receptor sequences between tumor infiltrating lymphocytes and T cells from ascites [100]. In a study comparing T cell populations in OC patients’ peripheral blood mononuclear cells and ascites, the proportion of CD3+ T-cells was increased in ascites [109]. Moreover, the CD4+/CD8+ ratio in ascites was decreased. Up to 27% of CD4+ cells were FoxP3-positive, i.e., significantly higher than in patients’ blood [109]. CD4+FoxP3+ Tregs were found to be attracted to ascites in a CCR4-dependent manner [107]. A higher level of CCR4 was found in naive CD4+ T-cells and resting Tregs from ascites, compared to that from PBMCs [109].

## 7. Conclusions

The development of peritoneal dissemination is a very complex process involving multiple cellular and acellular components. CAFs promote the remodeling of the extracellular matrix in ascitic fluid and activate cadherins and intergrins on tumor cells, which play the most significant role in cell–cell interactions. Both CAFs and TAMs can protect ovarian cancer cells from anoikis and can form the core of tumor spheroids. However, the specific mechanisms of these processes are still unknown. TAMs in ascites are polarized into the M2 phenotype by expressing M2 markers, mainly CD163 and CD206, and promote tumor cell invasion and chemoresistance. TAM interaction with other immune cells results in the development of immunosuppression in OC ascites. CAFs and TAMs are also essential for the peritoneal metastasis due to their assistance in the attachment of tumor cells to metastatic sites. The main function of T cells in ascites is supporting an immunosuppressive microenvironment.

In conclusion, the cell composition of ovarian cancer ascites is very inconsistent. Whether it depends on the clinicopathological characteristics of a patient’s tumor or not is unknown. The precise role of stromal and immune cells is also not yet fully understood. Finally, there is no clear evidence on the mechanism of tumor spheroid formation and spheroid composition. All these issues require in-depth investigations.

## Abbreviations

α-SMA	Alpha-Smooth Muscle Actin
CAF	cancer-associated fibroblast
ECM	extracellular matrix
EGF	epidermal growth factor
EMT	epithelial-mesenchymal transition
HGSOC	high grade serous ovarian carcinoma
MA	malignant ascites
MET	mesenchymal-epithelial transition
MS	milky spot
OC	ovarian cancer
OS	overall survival
PFS	progression-free survival
TAM	tumor-associated macrophage
TME	tumor microenvironment
VEGF	vascular endothelial growth factor
